# Impact of Sex and Age on mRNA COVID-19 Vaccine-Related Side Effects in Japan

**DOI:** 10.1128/spectrum.01309-22

**Published:** 2022-10-31

**Authors:** Masahiko Mori, Aiko Yokoyama, Ayami Shichida, Kimiko Sasuga, Takafumi Maekawa, Tadayoshi Moriyama

**Affiliations:** a Department of Internal Medicine, Sasebo Memorial Hospital, Sasebo, Nagasaki, Japan; b Regional Medical Cooperation Office, Sasebo Memorial Hospital, Sasebo, Nagasaki, Japan; c Medical Administration Division, Sasebo Memorial Hospital, Sasebo, Nagasaki, Japan; d Department of Medical Information, Sasebo Memorial Hospital, Sasebo, Nagasaki, Japan; e Department of Surgery, Sasebo Memorial Hospital, Sasebo, Nagasaki, Japan; f Department of Surgery, Fukuoka Central Hospital, Fukuoka, Fukuoka, Japan; g Department of Neurosurgery, Sasebo Memorial Hospital, Sasebo, Nagasaki, Japan; University of Georgia

**Keywords:** age, COVID-19, sex, side effect, vaccine

## Abstract

mRNA COVID-19 vaccination was initiated worldwide in late 2020, and its efficacy has been well reported. However, studies about vaccine-related side effects are sparse. A total of 262 health care workers who received mRNA COVID-19 vaccine BNT162b2 were recruited, and their vaccine-related side effects were investigated. Impact of sex and age on the side effects was statistically analyzed. A higher number of vaccine-related side effects among females versus males was identified (median 3 versus 2, *P* < 0.05, after the first dose, and 5 versus 2.5, *P* < 0.01, after the second dose). General fatigue, headache, chills, and fever were the culprit adverse symptoms. In multivariate analysis, females had an increasing number of side effects after receiving their first (B = 0.7; 95% confidence interval [CI], 0.2 to 1.2) and second (B = 1.5; 95% CI, 0.7 to 2.2) vaccine doses compared to that of males. In age analysis, the older group (≥60 years old) had a lower number of side effects than the younger group (B = −0.5 with a 95% CI of −1.1 to −0.02 after the first vaccine dose, and B = −2.1 with a 95% CI of −2.9 to −1.2 after the second vaccine dose). Additionally, prolonged time to recovery was found among females (*P* = 0.003 after the first dose; *P* = 0.008 after the second dose). Specifically, symptoms of general fatigue, headache, itching, swelling at the injection site, and dizziness were the culprit symptoms affecting recovery time. Several cutaneous and membranous symptoms, including “COVID arm,” were identified among females. These results highlight the impact of sex and age on side effects from mRNA COVID-19 vaccine and will aid in creating a safer vaccine.

**IMPORTANCE** We demonstrate sex- and age-related impact on mRNA COVID-19 vaccine-related side effects, with a higher number and frequency of side effects and prolonged time to recovery in females compared to males and negative correlation between age and vaccine-related side effects. Identification of unique age- and sex-specific adverse symptoms will provide the opportunity to better understand the nature of sex- and age-associated immunological differences and develop safer and more efficacious vaccines.

## INTRODUCTION

Administration of the newly developed mRNA COVID-19 vaccines was initiated in late 2020 worldwide (1, 2). In Japan, a national COVID-19 vaccination program was initiated on 17 February 2021, with priority given to health care personnel. The BNT162b2 mRNA vaccine (Comirnaty) (Pfizer, New York, NY, USA; BioNTech, Mainz, Land Rheinland-Pfalz, Germany) was used initially, followed by the mRNA-1273 vaccine (COVID-19 vaccine Moderna) (Moderna, Cambridge, MA, USA). As of 31 March 2022, nearly 102 million people in Japan (81% of the population) received their first dose, and 100 million people (79% of the population) received their second dose (3).

Multiple studies have published data on vaccine efficacy. For example, Polack et al. reported 95% protection against COVID-19 infection after the two-dose regimen of BNT162b2 (1). Later Baden et al. reported 94.1% efficacy in preventing COVID-19 illness by mRNA-1273 (2). Conversely, information about vaccine side effects remains sparse and incomplete. For example, frequency of acute and severe side effects, such as anaphylactic shock, after the first vaccination has been reported (4, 5); however, information about more common side effects, including frequency, age, and sex differences, and longitudinal follow up is limited. Lack of this information poses a significant problem during the prevaccination interview when describing the vaccine to the general public. This may lead to hesitation regarding vaccination, resulting in a subsequent decrease of vaccine coverage, especially among the younger generation, including medical students (6).

Our objective here was to identify the impact of sex and age on side effects from mRNA COVID-19 vaccine in a cohort of 262 vaccinated adults from Japan.

## RESULTS

### Characteristics of the cohort.

Of the 262 vaccinated individuals, 208 (79%) were female and 54 (21%) were male (Table 1). Median age at enrollment was 46.5 years (interquartile range [IQR], 35 to 57). Median body temperature prior to vaccination was 36.4°C (IQR, 36.2 to 36.5). In terms of the immunologic background, 48 (18%) had history of immune response-related diagnosis or event. Of these, 33 (13%) had allergy diagnosis, 12 (4.6%) had collagen disease diagnosis, and 10 (3.8%) had a history of side effects from previous vaccinations. Eight (3.1%) had a history of seizures. Five (1.9%) individuals cancelled their second vaccination dose appointments due to concern for severe side effects.

**TABLE 1:** Characteristics of the cohort

Characteristic	No. (%)
Total participants	262 (100)
Sex (female)	208 (79)
Age	46.5 (35–57)^*a*^
Body temp (°C)	36.4 (36.2–36.5)^*a*^
Immunologic history	48 (18)
Allergy diagnosis	33 (13)
Collagen disease diagnosis	12 (4.6)
Side effects from prior vaccination	10 (3.8)
Seizure history	8 (3.1)

a Median (interquartile range) is shown.

### Higher number of vaccine-related mainly systemic side effects among females.

**FIG 1:**
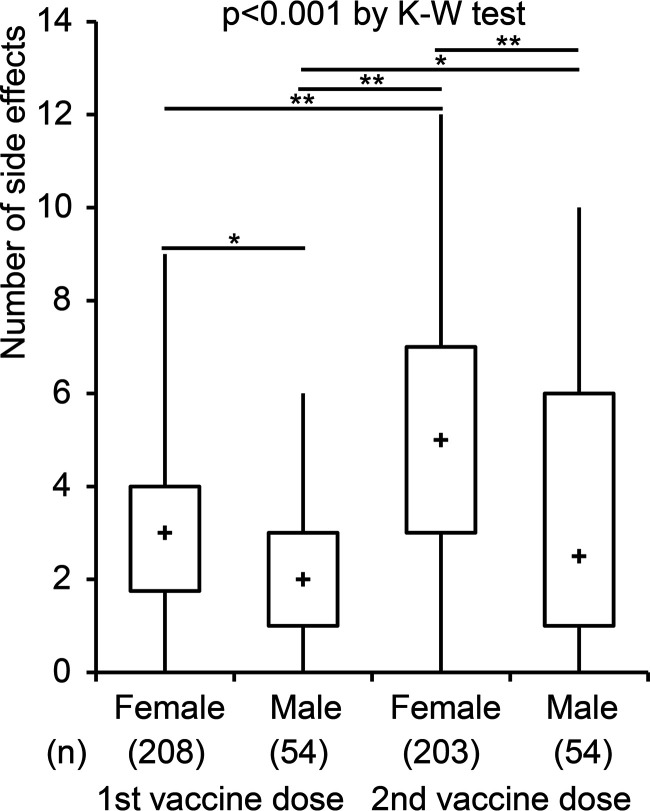
Differences in the number of side effects between females and males. Differences between females and males in the number of vaccine-related side effects by Kruskal-Wallis (K-W) test are shown (*P* < 0.001). Post-hoc analysis with Steel-Dwass test demonstrates significant differences between females and males after each vaccine dose as well as between first and second dose within each sex (*, *P* < 0.05; **, *P* < 0.01). The boxplot indicates minimum and maximum (line), 25th centile and 75th centile range (box), and median (plus sign within the box).

We first investigated whether the number of side effects was significantly different between females and males after the first and second dose (Fig. 1). Overall, the number of side effects was significantly different among the four groups (*P* < 0.001). There was a higher number of side effects in females than in males after each of the two vaccine doses as follows: median 3 versus 2, respectively, (*P* < 0.05) after the first vaccine dose, and 5 versus 2.5, respectively, (*P* < 0.01) after the second vaccine dose. Additionally, within each sex group, the number of vaccine-related side effects significantly increased after the second vaccine dose compared to that after the first vaccine dose as follows: median 3 versus 5, respectively, (*P* < 0.01) in females, and 2 versus 2.5, respectively, (*P* < 0.05) in males. These findings indicate that there is a significant difference in the number of vaccine-related side effects between females and males and that this difference strengthens after a repeat vaccination.

We next analyzed the differences between females and males in specific vaccine-related adverse symptoms (Table 2; see also Table S1 in the supplemental material). In total, we identified 30 symptoms related to the first vaccine dose and 39 symptoms related to the second vaccine dose. Of these, females experienced general fatigue (odds ratio [OR], 2.2; *P* = 0.03) and headache (OR, 6.1; *P* < 0.001) significantly more than the males after the first vaccine dose. After the second vaccine dose, females experienced general fatigue (OR, 3.5; *P* < 0.001), headache (OR, 5.7; *P* < 0.001), chills (OR, 2.3; *P* = 0.02), and fever (OR, 4.1; *P* < 0.001) significantly more frequently than males. These data suggest differences between females and males in developing certain COVID-19 vaccine-related adverse symptoms.

**TABLE 2:** Differences between females and males in frequency of vaccine-related side effects^*a*^

Dose and symptom	Sex	Symptom frequency (no.)^*b*^		OR^*c*^	*P* value
		+	−		
1st vaccine dose					
General fatigue	Female	75	133	2.2	0.03
	Male	11	43		
Headache	Female	55	153	6.1	<0.001
	Male	3	51		
2nd vaccine dose					
General fatigue	Female	150	53	3.5	<0.001
	Male	24	30		
Headache	Female	131	72	5.7	<0.001
	Male	13	41		
Chills	Female	81	122	2.3	0.02
	Male	12	42		
Fever (≥37.5°C)	Female	69	134	4.1	<0.001
	Male	6	48		

a Significant differences (*P* < 0.05 by Fisher’s exact test) in frequency of vaccine-related side effects are shown. Results for all side effects are shown in Table S1 in the supplemental material.

b +, Presence of a symptom; −, absence of a symptom.

c OR, odds ratio.

### Age-related differences in vaccine-related side effects.

**FIG 2:**
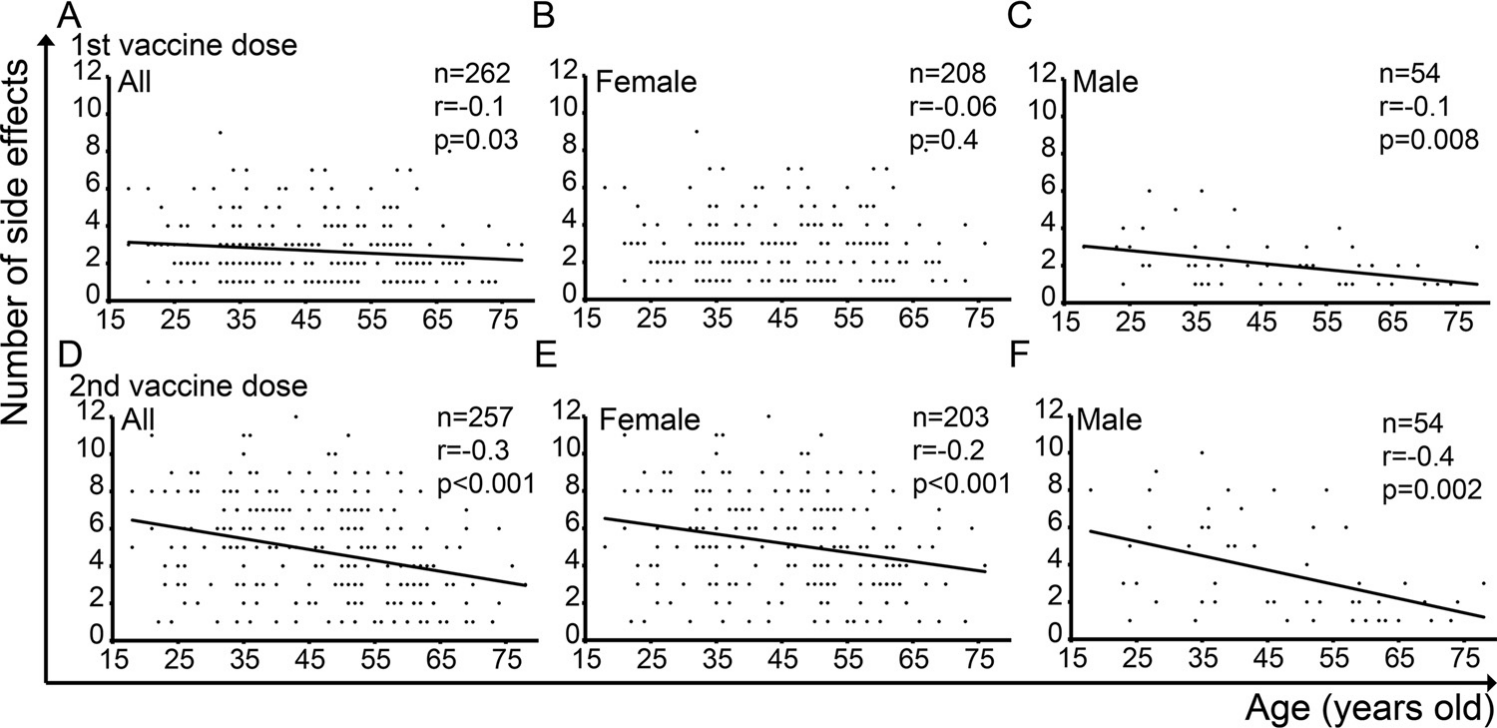
Correlation between age and number of vaccine-related side effects. Spearman’s correlation tests are shown.

To investigate the effect of age on vaccine-related side effects, we analyzed correlations between age and number of side effects. We found that in all study participants, there was a negative correlation between age and number of side effects after the first (*r* = −0.1; *P* = 0.03) and second vaccine dose (*r* = −0.3; *P* < 0.001) (Fig. 2A and D). Additionally, this negative correlation was observed individually among females after the second vaccine dose (r = −0.2; *P* < 0.001) (Fig. 2E) and males after the first (*r* = −0.1; *P* = 0.008) and second (*r* = −0.4; *P* = 0.002) (Fig. 2C and F) vaccine dose. The same trend toward negative correlation between age and number of side effects was observed among females after their first vaccine dose, although this did not reach significance (*r* = −0.06; *P* = 0.4) (Fig. 2B).

To investigate the impact of age on each symptom, we next analyzed the differences between the older group (age, ≥60 years old; *n* = 47 for the first vaccine dose and *n* = 46 for the second vaccine dose) and the younger group (age, ≤59 years old; *n* = 215 for the first vaccine dose and *n* = 211 for the second vaccine dose) in specific vaccine-related adverse symptoms (Table 3; see also Table S2 in the supplemental material). The younger group experienced side effects (OR, 5.0; *P* = 0.02) significantly more frequently than the older group after the first vaccine dose. After the second vaccine dose, the younger group experienced systemic symptoms of general fatigue (OR, 5.0; *P* < 0.001), headache (OR, 4.2; *P* < 0.001), joint pain (OR, 6.8; *P* < 0.001), chills (OR, 7.7; *P* < 0.001), fever (OR, 1.9; *P* = 0.03), and nausea (OR, 12; *P* = 0.001) significantly more frequently than the older group. These results suggest that age is an important factor contributing to the COVID-19 mRNA vaccine-related side effects.

**TABLE 3:** Differences in frequency of specific vaccine-related side effects between older group (age ≥60) and younger group (age ≤59)^*a*^

Dose and symptom	Age group	Symptom frequency (no.)^*b*^		OR^*c*^	*P* value
		+	−		
1st vaccine dose					
Any symptom	≤59	210	5	5.0	0.02
	≥60	42	5		
2nd vaccine dose					
General fatigue	≤59	157	54	5.0	<0.001
	≥60	17	29		
Headache	≤59	131	80	4.2	<0.001
	≥60	13	33		
Joint pain	≤59	96	115	6.8	<0.001
	≥60	5	41		
Chills	≤59	89	122	7.7	<0.001
	≥60	4	42		
Fever (≥37.5°C)	≤59	73	138	12	<0.001
	≥60	2	44		
Nausea	≤59	44	167	12	0.001
	≥60	1	45		

a Significant differences (*P* < 0.05 by Fisher’s exact test) in frequency of vaccine-related side effects are shown. Results for all side effects are shown in Table S2 in the supplemental material.

b +, Presence of a symptom; −, absence of a symptom.

c OR, odds ratio.

### Multivariate analysis: sex and age remained independent factors contributing to the number of vaccine-related side effects.

To confirm the impact of sex and age on vaccine-related side effects, we used multivariate analysis (Table 4). Both sex and age remained significant and independent factors affecting the number of vaccine-related side effects; for sex (females compared to males), B = 0.7 with a 95% confidence interval (CI) range of 0.2 to 1.2 after the first vaccine dose and B = 1.5 (95% CI, 0.7 to 2.2) after the second vaccine dose; for age (older group compared to younger group), B = −0.5 (95% CI, −1.1 to −0.02) after the first vaccine dose and B = −2.1 (95% CI, −2.9 to −1.2) after the second vaccine dose. Immunologic history was also identified as a significant factor after the first vaccine dose (B = 0.6 [95% CI, 0.1–1.1]), while seizure history did not have a significant effect after either vaccine dose. These results support the significant and independent effects of sex and age on the variety of COVID-19 vaccine-related side effects.

**TABLE 4:** Impact of sex and age on the number of vaccine-related side effects^*a*^

Dose and parameter	Univariate analysis		Multivariate analysis	
	B (95% CI)	*P* value	B (95% CI)	*P* value
1st vaccine dose				
All				
Sex (female)	0.8 (0.3–1.3)	0.003	0.7 (0.2–1.2)	0.006
Age (≥60 yrs old)	−0.6 (−1.1 to −0.03)	0.04	−0.5 (−1.1 to −0.02)	0.04
Body temp (°C)	0.3 (−0.3–0.9)	0.4		
Immunologic history	0.7 (0.1–1.2)	0.02	0.6 (0.1–1.1)	0.02
Seizure history	−0.05 (−1.3–1.2)	0.9		
Female				
Age (≥60 yrs old)	−0.5 (−1.1–0.2)	0.2		
Body temp (°C)	0.2 (−0.5–1.0)	0.5		
Immunologic history	0.7 (0.06–1.3)	0.03		
Seizure history	0.3 (−1.1–1.8)	0.6		
Male				
Age (≥60 yrs old)	−0.7 (−1.6–0.2)	0.1		
Body temp (°C)	−0.1 (−1.3–1.0)	0.8		
Immunologic history	0.3 (−0.9–1.4)	0.6		
Seizure history	−1.1 (−3.1–0.9)	0.3		
2nd vaccine dose				
All				
Sex (female)	1.6 (0.8–2.4)	<0.001	1.5 (0.7–2.2)	<0.001
Age (≥60 yrs old)	−2.3 (−3.1 to −1.5)	<0.001	−2.1 (−2.9 to −1.2)	<0.001
Body temp (°C)	1.3 (0.2–2.3)	0.02	0.7 (−0.4–1.7)	0.2
Immunologic history	0.6 (−0.3–1.4)	0.2		
Seizure history	−0.2 (−2.2–1.7)	0.8		
Female				
Age (≥60 yrs old)	−2.1 (−3.0 to −1.1)	<0.001	−1.9 (−2.9 to −0.9)	<0.001
Body temp (°C)	1.4 (0.2–2.6)	0.02	0.9 (−0.3–2.0)	0.1
Immunologic history	0.3 (−0.6–1.3)	0.5		
Seizure history	−0.7 (−2.9–1.5)	0.5		
Male				
Age (≥60 yrs old)	−2.6 (−4.3 to −1.0)	0.002		
Body temp (°C)	0.6 (−1.6–2.9)	0.6		
Immunologic history	1.0 (−1.2–3.2)	0.4		
Seizure history	1.5 (−2.4–5.4)	0.4		

a Linear regression model analyses are shown. Variables with significance (*P* < 0.05) in univariate analysis were applied to the multivariate analysis.

It is curious that, in females, age was not a significant factor affecting the number of vaccine-related side effects after the first vaccine dose (*r* = −0.06; *P* = 0.4) (Fig. 2B), while their immunologic history was significant (B = 0.2 [95% CI, 0.06 to 1.3]) (Table 4). These findings raise the possibility that one’s immunologic history may interfere with correlation between age and number of vaccine-related side effects.

### Longitudinal analysis: prolonged recovery rate from vaccine-related side effects in females due to key systemic symptoms.

**FIG 3:**
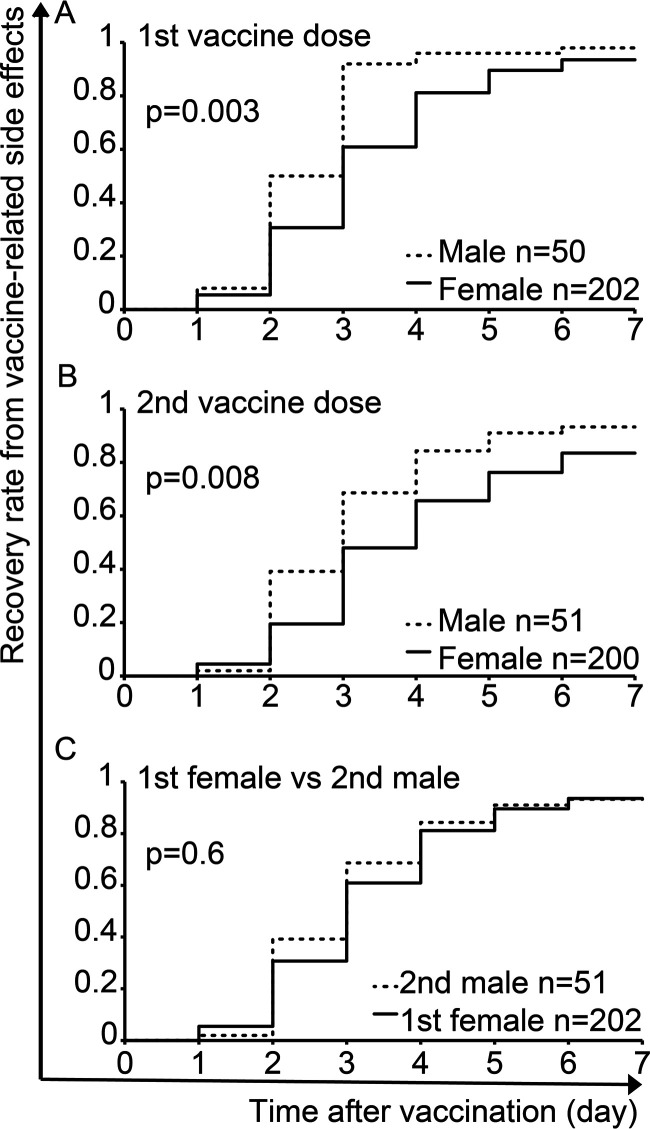
Differences between females and males in recovery rate from vaccine-related side effects after the first (A) and second (B) vaccine dose. (C) Differences in recovery rate between females after the first vaccine dose and males after the second vaccine dose. Log-rank tests are shown.

Next, we assessed the impact of sex on recovery rate from vaccine-related side effects among 252 out of 262 subjects who had adverse symptoms after the first vaccine dose and 251 out of 257 subjects after the second vaccine dose. The duration until full recovery from all vaccine-related side effects was a median of 3 (IQR, 2 to 4) days in females versus 2.5 (2 to 3) days in males (*P* < 0.001) after the first vaccine dose and 4 (3 to 5) days versus 3 (2 to 4) days (*P* = 0.001) after the second vaccine dose. We found that recovery rate from a side effect was significantly prolonged among females compared to males after each vaccine dose with a 94% recovery rate in females versus 98% in males (*P* = 0.003) after the first dose (Fig. 3A) and 83% versus 92%, respectively, (*P* = 0.008) after the second dose (Fig. 3B). These findings indicate differences between females and males in the rate of recovery from vaccine-related side effects.

However, there was no significant difference in the recovery rate from vaccine-related side effects between females after the first dose and males after the second dose (*P* = 0.6) (Fig. 3C). Progress of side effects that males suffered after the second vaccine dose may be similar to the progression of side effects that females already experienced after the first vaccine dose.

Additionally, we examined the impact of antipyretic treatment on side effect progression. Seventeen out of 252 (6.7%) subjects after the first vaccine dose and 131 out of 251 (52%) subjects after the second vaccine dose received antipyretics. Interestingly, these subjects who received antipyretics had a significantly longer time until their recovery from symptoms compared to the subjects without antipyretic treatment as follows: median 4 days versus 3 days (*P* = 0.02) after the first vaccine dose and median 4 days versus 3 days (*P* < 0.001) after the second vaccine dose. Since medical treatment with antipyretics was started after the diagnosis of symptoms, subjects treated with antipyretics would have more severe symptoms and subsequently longer time until their recovery.

We next analyzed individual symptoms affecting recovery rate (Table 5; see also Table S3 and Table S4 in the supplemental material). Among all groups, symptoms of general fatigue (adjusted hazard ratio [aHR], 0.7; *P* = 0.03), headache (aHR, 0.7; *P* = 0.02), and itching (aHR, 0.3; *P* = 0.03) were identified as significant symptoms, contributing to the prolonged recovery after the first vaccine dose. After the second vaccine dose, swelling at the injection site (aHR, 0.7; *P* = 0.03), general fatigue (aHR, 0.6; *P* = 0.02), headache (aHR, 0.7; *P* = 0.03), and dizziness (aHR, 0.4; *P* = 0.04) significantly prolonged time to recovery. Separate analysis within each sex group demonstrated that, in females, general fatigue (aHR, 0.6; *P* = 0.02) after the first vaccine dose and swelling at the injection site (aHR, 0.7; *P* = 0.03) and dizziness (aHR, 0.3; *P* = 0.03) after the second vaccine dose significantly contributed to the prolonged recovery. In males, no symptom played a significant role in the recovery rate after the first vaccine dose, while general fatigue (aHR, 0.4; *P* = 0.01) significantly prolonged recovery rate after the second vaccine dose. These data point out specific symptoms as the main culprits in prolonging the recovery rate from vaccine-related adverse effects.

**TABLE 5:** Symptoms playing the key role in recovery rate from COVID-19 vaccine-related adverse effects^*a*^

Dose and symptom	HR^*b*^ (95% CI)	*P* value	aHR^*c*^ (95% CI)	*P* value
1st vaccine dose				
All				
General fatigue	0.5 (0.4–0.7)	<0.001	0.7 (0.5–0.9)	0.03
Headache	0.5 (0.4–0.7)	<0.001	0.7 (0.5–0.9)	0.02
Chills	0.6 (0.3–0.9)	0.03	0.8 (0.5–1.4)	0.4
Nausea	0.5 (0.3–0.9)	0.04	0.7 (0.4–1.3)	0.3
Itching	0.3 (0.1–0.9)	0.04	0.3 (0.09–0.9)	0.03
Female				
General fatigue	0.5 (0.4–0.7)	<0.001	0.6 (0.5–0.9)	0.02
Headache	0.6 (0.4–0.8)	0.001	0.8 (0.6–1.2)	0.3
Chills	0.6 (0.3–0.9)	0.04	0.8 (0.5–1.5)	0.5
Nausea	0.5 (0.3–0.9)	0.04	0.7 (0.3–1.3)	0.2
Diarrhea	0.5 (0.2–0.9)	0.04	0.7 (0.3–1.6)	0.2
Male				
No symptoms with significance in univariate analysis				
2nd vaccine dose				
All				
Swelling at injection site	0.6 (0.5–0.8)	0.002	0.7 (0.5–0.9)	0.03
General fatigue	0.4 (0.3–0.6)	<0.001	0.6 (0.5–0.9)	0.02
Headache	0.5 (0.4–0.7)	<0.001	0.7 (0.5–0.9)	0.03
Muscle pain	0.8 (0.6–0.9)	0.04	0.9 (0.7–1.2)	0.6
Joint pain	0.7 (0.5–0.9)	0.005	0.9 (0.7–1.4)	0.9
Chills	0.6 (0.5–0.8)	0.002	1.1 (0.7–1.6)	0.7
Fever (≥37.5°C)	0.6 (0.4–0.8)	0.001	0.8 (0.6–1.2)	0.3
Nausea	0.6 (0.4–0.8)	0.004	0.8 (0.5–1.2)	0.2
Dizziness	0.3 (0.1–0.8)	0.02	0.4 (0.2–0.9)	0.04
Female				
General fatigue	0.5 (0.4–0.7)	<0.001	0.7 (0.5–1.1)	0.1
Headache	0.6 (0.4–0.8)	0.002	0.8 (0.5–1.1)	0.1
Swelling at injection site	0.7 (0.5–0.9)	0.01	0.7 (0.5–0.9)	0.03
Joint pain	0.7 (0.5–0.9)	0.03	0.8 (0.6–1.2)	0.4
Chills	0.7 (0.5–0.9)	0.03	1.2 (0.8–1.9)	0.4
Fever (≥37.5°C)	0.7 (0.5–0.9)	0.02	0.9 (0.6–1.3)	0.5
Nausea	0.6 (0.4–0.9)	0.006	0.7 (0.4–1.0)	0.07
Dizziness	0.3 (0.1–0.8)	0.02	0.3 (0.1–0.9)	0.03
Male				
General fatigue	0.3 (0.2–0.6)	0.001	0.4 (0.2–0.8)	0.01
Headache	0.4 (0.2–0.9)	0.02	0.7 (0.3–1.7)	0.5
Chills	0.4 (0.2–0.9)	0.03	0.7 (0.3–1.6)	0.4

a Cox hazard model analyses of recovery rate for each adverse symptom are shown. Symptoms with significance (*P* < 0.05) in univariate analysis were applied for multivariate analysis. Results for all symptoms are shown in Table S3 in the supplemental material.

b HR, hazard ratio.

c aHR, adjusted hazard ratio.

### Long-lasting cutaneous and membranous vaccine-related symptoms, including “COVID arm.”

**FIG 4:**
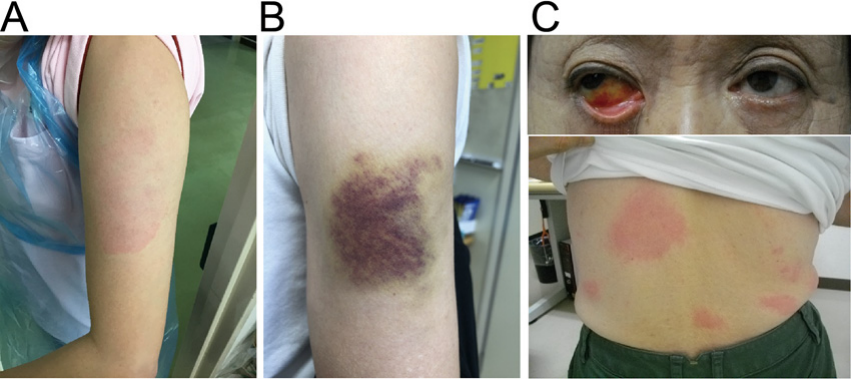
Cutaneous and membranous symptoms after COVID-19 vaccination. A 38-year-old female with erythema annulare at the injection site (A), 62-year-old female with bruising at the injection site (B), and 69-year-old female with conjunctiva icterus and bleeding and erythema figuratum at body trunk (C). These symptoms were identified after the second vaccine dose. Images were acquired on day 7 after vaccination.

Finally, we noticed several cutaneous and membranous symptoms that lasted more than 1 week after vaccination, although they did not reach statistical significance. Examples include a 38-year-old female with erythema annulare at the injection site (Fig. 4A), a 62-year-old female with bruising at the injection site (Fig. 4B), and a 69-year-old female with conjunctival icterus and bleeding and erythema figuratum (Fig. 4C). These symptoms were identified after the second vaccine dose, and images were taken on day 7 after the vaccination. These findings suggest that a few cutaneous or membranous symptoms require a longer time to recovery without categorizing them.

## DISCUSSION

This study systematically investigated the impact of sex and age on vaccine-related side effects in a cohort of COVID-19 mRNA-vaccinated adults. Here, using cross-sectional and longitudinal analyses, we identified a significantly higher frequency of several vaccine-related adverse symptoms and a prolonged recovery rate in females compared to that in males. Additionally, we found a negative correlation between age and the number of side effects.

Various sex differences in disease progression, medication efficacy, and medication- or vaccine-related side effects have been reported and summarized (7). For example, in collagen diseases, higher frequency of disease in females compared to that in males was reported for Sjögren’s syndrome (9:1), systemic lupus erythematosus (8:2), rheumatoid arthritis (7:3), and multiple sclerosis (6:4) (8). In cancer, males show increased disease susceptibility to nonreproductive cancers, including lung, larynx, and bladder cancer, compared to that in females (7, 9). Regarding treatment-related sex differences, newly developed immune checkpoint inhibitors (for example, ipilimumab, tremelimumab, nivolumab, and pembrolizumab) were shown to be more effective in males than in females against advanced or metastatic cancers, including melanoma and lung cancer (10). Furthermore, chemotherapy- (11–13) and immunotherapy-related (14) systemic side effects have been described more frequently in females than in males.

In this study, a higher frequency of COVID-19 vaccine-related adverse symptoms, such as general fatigue, headache, chills, and fever, in females compared to that in males was identified. This was consistent with previous cross-sectional studies (15, 16). Moreover, our longitudinal analyses identified longer recovery from side effects in females compared to that in males after each vaccine dose. Additionally, several findings suggested that males after the second vaccine dose were somewhat comparable and not significantly different from females after the first vaccine dose in terms of the number of side effects (*post hoc* analysis in Fig. 1), recovery rate (Fig. 3C), and general fatigue, which was the significant key symptom contributing to the decreased recovery rate (Table 5). Overall, these findings hint at the possibility that females at baseline may have a stronger immune system compared to males. The precise mechanism mediating these sex differences is not completely understood, but the effects of sex hormones on immune system are being investigated (7). Stimulation of humoral and cellular-mediated immune responses by estrogen (17–20) and progesterone (21) and stimulation of anti-inflammatory responses by androgens, including testosterone (22), have been described. The effects of sex hormones on immune cells may be contributing to the differences of COVID-19 vaccine-related side effects in females and males. In support of this notion is the recent study reporting a higher frequency of myocarditis or pericarditis after COVID-19 vaccine in young males compared to females (23).

Our findings of age-related differences in side effects following the COVID-19 vaccine (negative correlation between age and number of COVID-19 vaccine-related side effects and higher frequency of local and systemic symptoms after the first and second vaccine doses, respectively, in younger people) are curious. It may be related to the decline in immune function with age, referred to as “immunosenescence” (24). Immunosenescence affects both cellular-mediated and humoral immunity. Thymic involution is one of the significant age-related changes, resulting in a decrease of naive T-cell production and subsequent decline of immune response against novel antigens (25–27). Individual immunologic background, such as history of collagen disease, allergy, and side effects to prior vaccination may interfere the impact of age on vaccine-related side effects, particularly as we showed in females after the first vaccine dose (Fig. 2B and Table 4). Such differences in the immune response of hosts, depending on their immunologic background, have been reported in HIV infection, with acceleration of immunosenescence in HIV-positive individuals compared to HIV-negative individuals of the same sex and age (28). While this impact of an individual’s immunological background on vaccine-related side effects contributes significantly to the postvaccination adverse symptoms at the time of the first vaccine dose, it loses its significance as a variable after the second vaccination (Table 4). These notions underscore the importance of obtaining an accurate history about an individual’s immunological background at a hospital or a vaccine venue, especially prior to the first vaccination.

Several long-lasting cutaneous and membranous vaccine-related symptoms were identified in this study after the second vaccine dose (Fig. 4). Among them, erythema annulare at the injection site (Fig. 4A), referred to as “COVID arm,” was previously reported (29). Delayed hypersensitivity reaction by type IV allergic response has been proposed as the mechanism of erythema annulare, with perivascular infiltration of lymphocytes and eosinophils (30, 31). Although this cutaneous symptom was more common after mRNA-1273 (COVID-19 vaccine Moderna) vaccination, our study demonstrates its association with the BNT162b2 COVID-19 vaccine as well.

In addition to the unique symptoms related to the side effects following the COVID vaccine, the higher frequency of side effects due to mRNA vaccines versus other vaccines is also considered. The U.S. CDC previously reported that the frequency of severe side effects, including anaphylactic reaction, caused by mRNA vaccines was higher than that caused by non-mRNA vaccines. For example, 11.1 cases per million after the first dose of BNT162b2 and 2.5 cases per million after the first dose of mRNA-1273, which was 2 to 8.5 times higher than the frequency for all other non-mRNA vaccines (1.31 cases per million doses) (32, 33). mRNA vaccines contain unique components such as polyethylene glycol, which works as a surfactant of mRNA and supports stability and transport of mRNA. Side effects caused by such unique mRNA vaccine properties should be considered, and our age- and sex-related side effect information from the novel vaccine may be helpful for understanding the mechanism of side effects and future safer vaccine development (33–35).

Introduction of Cox proportional hazard model analysis for longitudinal analysis may be considered a limitation in this study. The duration was set from vaccination to being free from symptoms, and Cox proportional hazard model analysis was introduced. To prevent violation of proportional hazard assumption, especially considering the side effect symptom-free time after vaccination, analysis by the time-dependent Cox proportional hazard model would be ideal. However, since some symptoms were seen intermittently or identified and diagnosed after a follow up with doctors (for example, lymph node swelling and intraoral lesions), accurate onset day of symptoms for the time-dependent Cox proportional hazard model analysis was not available. Further analyses would be warranted especially for the longitudinal evaluation of vaccine side effects.

In conclusion, this study investigated the impact of sex and age on mRNA COVID-19 vaccine-related side effects in vaccinated adults. We found that vaccine-related side effects are more frequent among females and that females have a decreased recovery rate compared to that of males. We showed the decrease of the number of side effects with age and investigated the important question of an individual’s immunologic background prior to the first COVID-19 vaccine dose. Our results are consistent with previous studies demonstrating sex and age differences on generation of immune responses, treatment efficacy, and treatment side effects in a variety of conditions. Identification of unique age- and sex-specific adverse symptoms will provide the opportunity to better understand the nature of sex- and age-associated immunological differences and develop safer and more efficacious vaccines.

## MATERIALS AND METHODS

### Subjects and data collection.

This research was approved by the ethical review boards at Sasebo Memorial Hospital, Japan (approval number 202107). All participants provided written informed consent for the collection of information about side effects and subsequent analysis. A total of 262 hospital employees who received the BNT162b2 COVID-19 vaccine (Cominarty) (Pfizer, New York, NY, USA; BioNTech, Mainz, Land Rheinland-Pfalz, Germany) for both first and second doses were recruited from March 2021 to August 2021. None of the study participants had a history of COVID-19 diagnosis prior to vaccination. In this study, we defined the effect of sex and age on the number and duration of side effects as the primary endpoint. The effect of each symptom on the duration of side effects after vaccination was set as the secondary endpoint. Duration of a side effect was defined as the time to be free from all symptoms after vaccination. Background information, such as body temperature prior to vaccination, past immunologic history (for example, diagnosis of allergy or collagen disease or side effects from prior vaccination), and seizure history, was collected using the national Pre-vaccination Screening Questionnaire for COVID-19 vaccine form, issued by the Japanese Ministry of Health, Labor and Welfare (see Fig. S1 in the supplemental material). This information was used for covariate analysis. Patients were interviewed by medical doctors in an outpatient setting within 1 week after each vaccination. Side effects that occurred during that postvaccination week were discussed, photographed when necessary, treated, and documented in the electronic medical chart. All of the side effects and their frequencies are documented in Table S1 in the supplemental material.

### Statistical analysis.

Statistical analysis was performed using SPSS 21.0 (IBM, Armonk, NY, USA). The effect of sex (female versus male) or vaccine dose (first versus second dose) on the number of side effects was tested by Kruskal-Wallis test with Steel-Dwass test for *post hoc* analysis. Sex differences in frequency of clinical symptoms were analyzed by Fisher’s exact test. Spearman’s correlation test was used for correlation between age and number of side effects. Age differences in frequency of clinical symptoms were analyzed by Fisher’s exact test between the older group (age, ≥60 years old) and the younger group (age, ≤59 years old). Linear regression model with multivariate analysis was used to evaluate the impact of sex and age on the variety of side effects. In a longitudinal analyses, log-rank test was performed to assess sex differences in recovery rate from the vaccine-related side effects. The Cox hazard model was applied to establish the side effects that affect the recovery rate.
