# Supplementary material for: Impact of Sex and Age on mRNA COVID-19 Vaccine-Related Side Effects in Japan
Source: Microbiol Spectr. 2022 Oct 31;10(6):e01309-22. doi: 10.1128/spectrum.01309-22 (PMC9769945; doi:10.1128/spectrum.01309-22)
Supplement: Supplemental file 1 — Fig. S1. Download spectrum.01309-22-s0001.pdf, PDF file, 0.2 MB [file spectrum.01309-22-s0001.pdf]

Prevaccination Screening Questionnaire for COVID-19 vaccine

*Please fill in or check the ☑ boxes inside the bold frame

注意
本予診票を用いて請求を行うことはできません。
日本語の予診票に転記の上、請求を行ってください。

Address on the resident card	Prefecture		City			
	Address					
Furigana		Tel. No.	( ) -			
Name						
Date of birth	Year Month Day	(years old)	□ male □ female	Body temperature before examination	Degrees	

Question	Response field		Field filled in by doctor
Are you receiving the COVID-19 vaccine for the first time?<br>(If you have been vaccinated before, date of 1st time: MM/ DD, date of 2nd time: MM/ DD)	☐ yes	☐ no	
Is the city, town, or village where you currently reside the same as the city, town, or village stated on the coupon?	☐ yes	☐ no	
Have you read the "Instructions for the COVID-19 vaccine" and do you understand the effects and adverse side effects?	☐ yes	☐ no	
Do you fall into one of the target groups that have a higher priority for this vaccine?<br>☐ Medical personnel, etc. ☐ Person 65 years or older ☐ Person 60 to 64 years old ☐ Worker at a senior citizen facility, etc.<br>☐ Person with an underlying disease (name of disease:)	☐ yes	☐ no	
Are you currently suffering from any kind of illness and receiving treatment or medication?<br>Name of disease: ☐ heart disease ☐ kidney disease ☐ liver disease ☐ blood disease ☐ disease that makes it difficult to stop bleeding ☐ immune deficiency<br>☐ other ( )<br>Nature of treatment: ☐ blood-thinning medicine ( ) ☐ other ( )	☐ yes	☐ no	
Have you had a fever or gotten sick in the last month? Name of disease ( )	☐ yes	☐ no	
Are there any parts of your body that are not feeling well today? Condition ( )	☐ yes	☐ no	
Have you ever had a convulsion (seizure)?	☐ yes	☐ no	
Have you ever experienced severe allergic symptoms (such as anaphylaxis) from medications or foods?<br>Medication or food that caused the problem ( )	☐ yes	☐ no	
Have you ever been sick after receiving a vaccine?<br>Type of vaccine ( ) Condition ( )	☐ yes	☐ no	
Is there any possibility that you are currently pregnant (for example, your period is later than expected)? Or are you breastfeeding?	☐ yes	☐ no	
Have you had any vaccines within the last two weeks?<br>Type of vaccine ( ) Date of vaccine ( )	☐ yes	☐ no	
Do you have any questions about the vaccine today?	☐ yes	☐ no	

Field filled in by doctor	In light of the results of the questions above and examination, today's vaccine is (□ possible, □ not possible).<br>I have explained the effects of the vaccine, side effects, and the Relief System for Injury to Health with Vaccination to the patient.<br>□ The person to be vaccinated is under 6 years old (fill in if applicable)	Signature and seal of doctor

COVID-19 Vaccination Request Form

After receiving a medical examination and explanation from a doctor and understanding the effects and side effects of the vaccine, do you wish to receive this vaccine?
(□ I wish to be vaccinated/ □ I do not wish to be vaccinated)

The purpose of this preliminary medical examination form is to ensure the safety of the vaccine.
I understand this and consent to this prevaccination Screening Questionnaire being submitted to the municipal government, the All-Japan Federation of National Health Insurance Organizations, and the National Health Insurance Organization.

Date: Signature of vaccinated person or their guardian

(*If the person to be vaccinated is unable to sign the form by himself/herself, a representative must sign the form, and the representative's name and relationship to the person to be vaccinated must be indicated.)
(*In the case of a person under 16 years of age, the form must be signed by the guardian; in the case of an adult ward, the form must be signed by the person himself/herself or the adult guardian.)

Field filled in by doctor	Name of vaccine and lot number	Inoculation amount	Vaccination location, name of doctor, and date of vaccination	*Please fill in the medical institution code and vaccination date so that they fit within this field.
	Seal position<br>*Paste it straightly along with the frame.<br>(Note: Make sure that the expiration date has not expired.)	ml	Vaccination location<br>Name of doctor	Medical institution code<br>Date of vaccination *Example: April 1, 2021 →2021/04/01
